# Different planning policies for the initial movement velocity depending on whether the known uncertainty is in the cursor or in the target: Motor planning in situations where two potential movement distances exist

**DOI:** 10.1371/journal.pone.0265943

**Published:** 2022-03-30

**Authors:** Ryoji Onagawa, Kae Mukai, Kazutoshi Kudo

**Affiliations:** 1 Laboratory of Sports Sciences, Department of Life Sciences, Graduate School of Arts and Sciences, The University of Tokyo, Tokyo, Japan; 2 Research Fellow of Japan Society for the Promotion of Science, Tokyo, Japan; 3 Faculty of Science and Engineering, Waseda University, Tokyo, Japan; University of Münster, GERMANY

## Abstract

During goal-directed behaviors, individuals can be required to start a movement before deciding on the final goal. Previous studies have focused on the initial movement direction in situations involving multiple targets in different directions from the starting position and have shown that the movement is initiated in the average direction among the target directions. However, the previous studies only included situations with targets at equivalent distances, and the characteristics of motor planning in situations with multiple movement possibilities over different potential distances are unclear. In such situations, movement velocity is another important control variable. Furthermore, while previous studies examined situations with an uncertain motor target position, uncertainty can also exist in the effector position (e.g., body or tool locations). Therefore, we examined (1) whether the average output is confirmed in the initial movement velocity during execution in situations involving two potential movements with different distances. In addition, we examined (2) whether planning of the movement velocity can differ depending on the presence of uncertainty in the cursor or the target. In the main conditions, the participants were required to start a reaching movement with two potential movement distances; in the *two-cursor* condition, two cursors were presented before the start of the trial, and in the *two-target* condition, two targets were presented. As a control condition, a distance condition corresponding to each main condition was also performed. In the control condition, the initial movement velocity varied linearly with distance. Then, we tested whether the initial movement velocity in situations with two potential movement distances would follow the averaging output of the corresponding control condition. The results revealed that while the initial movement velocity in the *two-target* condition was slower than the averaging output, that in the *two-cursor* condition approached the averaging output. These results suggest that the velocity profile of the goal-directed movement is not simply averaged in a situation where two potential targets exist, and that there is a difference in the planning policy of the initial movement depending on whether the known uncertainty is for the movement goal or the effector.

## Introduction

Humans often face situations in which movements must be initiated in uncertain future states. Motor planning in conditions with multiple potential targets has been investigated using a go-before-you-know paradigm [1–12], in which an individual is simultaneously presented with multiple potential targets and is required to launch a goal-directed reaching movement toward the competing target before knowing the final target location, which is cued after movement onset. Many previous studies using such tasks reported that humans frequently generate movements in the average direction between potential target locations and the correct target direction after obtaining information about the final target location [1,2,5,7,13–16]. Additionally, the averaging behavior has been confirmed not only in relation to basic movement-related variables such as direction and orientation of the reaching movements, but also in the specification of a sensorimotor control policy that sets feedback gains to determine how the motor system responds to errors induced by noise or external perturbations [8].

How averaging behavior is taken under several task setting under uncertainty about the final target still under the debate. Previous studies proposed two explanations. The first explanation is that averaging behavior is due to the competition between multiple reach plans towards the potential targets [2,5,14]. This explanation was supported by the neurological evidence of the time in which the motor system simultaneously represents competing motor plans [17,18]. The other explanation is the averaging behavior is due to actual planning of such a trajectory. Recent studies have shown that such averaging behavior likely occurs as it is the more ideal response given task uncertainty. In cases where the average response is not optimal, it is not generated [4,9–11]. In line with this idea, neural evidence has recently confirmed that dorsal premotor cortex represents only a single movement in the presence of competing movements [19].

Although many studies have consistently reported this averaging behavior, the situations examined in these studies were limited to cases where the distances between the movements were relatively comparable. However, in daily life, situations involving multiple potential actions with equal movement distances are much fewer than those involving multiple actions with different movement distances, and motor planning in the presence of multiple movements with different distances simultaneously is unclear. In such situations, the movement velocity is an important control parameter because it varies depending on the required movement distance [20–22]. Even in motor planning considering multiple potential movements with different distances, if multiple reach plans towards potential targets are to be averaged, the initial movement velocity may approach the average.

On the other hand, it is possible that the averaging behavior may not be followed. Recent studies confirmed responses that do not follow averaging behavior, and the common findings in those studies are that deviations from the averaging behavior occur when averaging offers no benefit in terms of energetic cost [3,4,11] or task optimization [9,10]. Similarly, in motor planning under multiple potential goals with different distances, deviation from the averaging behavior may occur from the perspective of biomechanical cost. In a reaching movement, rapid acceleration and deceleration during movements involve a biomechanical cost, just as repeated rapid acceleration and deceleration in an automobile reduces fuel efficiency. The averaging behavior always involves a corrective cost since it requires acceleration when the far target is correct and deceleration when the near target is correct. In addition, when the movement is initiated at a fast velocity corresponding to the movement toward the farther target, a rapid deceleration is required when a nearer target is correct, resulting in a large corrective cost. In contrast, if the movement starts at a weaker velocity, the nearby target can be reached without any change, and the far target can be reached by accelerating during the movement. In this case, acceleration in a moving state has a smaller cost than deceleration and considering the irreversibility of movement, there is more time margin for correction. Therefore, it is conceivable that initiation of a movement at a velocity corresponding to a single reach to the near target may be a more desirable strategy.

It is also unclear how the locus of uncertainty affects motor planning. While the uncertainty of the target is a critical issue, the simultaneous uncertainty of the effector (e.g., body locations) is another critical point. Individuals are required to execute movements without knowing the exact positions of body parts and tools, as well as the target locations. Even with a known uncertainty in either the effector or the target position, if the vector from the starting position to the target is the same, it can be reached with the same motor output. However, different sensory modalities for the perception of position are involved between the effector and the movement target. While visual information is crucial in the perception of the target location [23], the perception of the effector location involves proprioceptive information, efferent neural signals, and visual information [23]. Moreover, perturbations or changes in the effector location during a movement need to be detected continuously, while perturbations or changes in the target location can be detected as a more static change in the global coordinates. The difference between the effector and target in position estimation may alter the determination of the initial movement velocity, and the approach to deal with uncertainty in movement targets may be different from the approach to deal with uncertainty in body position.

**Fig 1:**
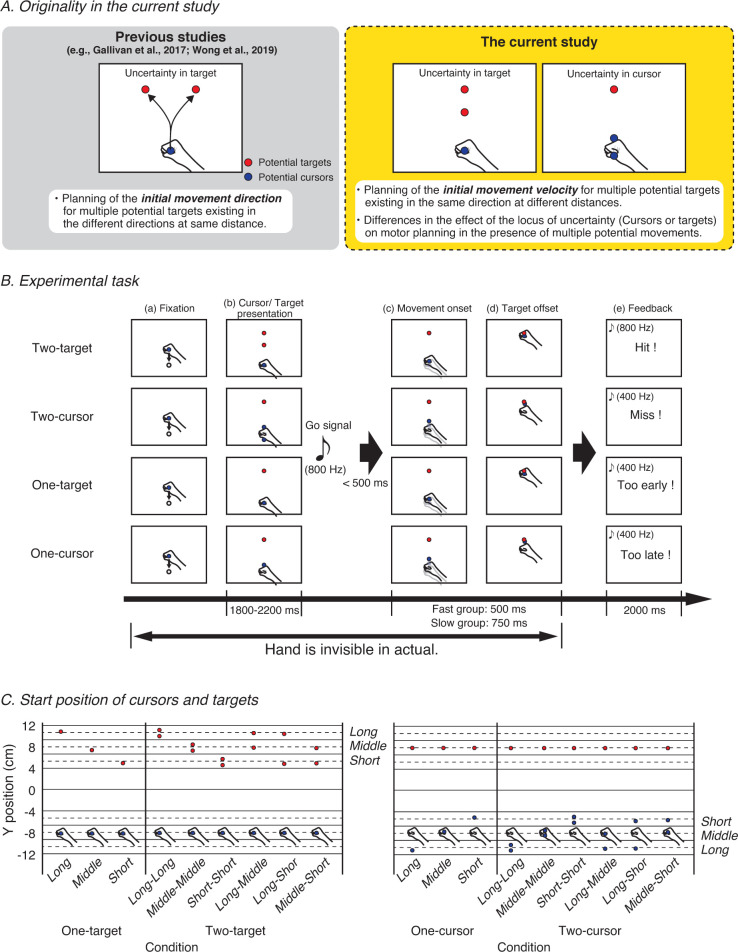
Experimental setting. (A) Originality in the current study. (B) Experimental task. The study used four conditions for the initial state of cursor and target: the *two-target* condition, the *two-cursor* condition, the *one-target* condition, and the *one-cursor* condition. In the *two-target* condition, at the beginning of the task, a cursor and two potential targets were presented along a vertical line. In this condition, after movement onset, one of the two targets disappeared, and the other remained at the initial position. The participants were required to reach and keep the cursor on the remaining target within a time constraint (500 or 750 ms in the fast and slow groups, respectively). In the *two-cursor* condition, at the beginning of the task, two potential cursors and a target were presented along a vertical line. In this condition, after movement onset, one of the two cursors disappeared, and the other remained at the initial position. The *one-target* condition and the *one-cursor* condition were the control conditions for the *two-target* and *two-cursor* conditions, respectively. (C) Start positions of cursors and targets. In the analyses, the distance between the cursor and the target was classified into three categories: long (L: 18–22 cm), middle (M: 14–18 cm), and short (S: 10–14 cm), starting with the farthest one. The probability of appearance of these areas was set to be equal. Thus, the *one-target* and *one-cursor* conditions included the three distance conditions, L, M, and S, with 16 trials performed for each condition. However, the *two-target* and *two-cursor* conditions included six distance conditions, namely, LL, MM, SS, LM, LS, and MS, each of which was also assessed over 16 trials.

Therefore, we examined two main issues in this study (Fig 1A). First, we examined (1) whether averaging behavior is confirmed in the initial movement velocity during the execution in situations with two potential movements with different distances. In addition, the current study examined (2) whether the planning of the movement velocity differed depending on the presence of uncertainty in the cursor or in the target. In addition, to investigate whether time constraints affect behavior in the above perspective, participants were divided into two time constraint groups.

## Methods

### Participants

Twenty-two right-handed neurologically healthy adults (age: 22.0 ± 2.6 years, 10 men) participated in this study. All patients had normal or corrected-to-normal vision. All participants were naive to the purposes of this study and provided written informed consent. This study was approved by the Ethics Committee of the Graduate School of Arts and Sciences, University of Tokyo. All experimental procedures adhered to approved guidelines. Informed consent was obtained from each participant before the experiments in a written format.

### Experimental setup

The participants sat in a quiet, dim room. A pen tablet with sufficient workspace to measure the subjects’ arm reach movement (Intuos 4 Extra Large, workspace: 488 × 305 mm; Wacom) was set on the table. A monitor (KH2500V-ZX2, 24.5 inches, 1920 × 1080 pixels, vertical refresh rate, 240 Hz; I-O DATA) that was used to present stimuli was set with an approximately 30° gradient angle over the pen-tablet. The participants manipulated a cursor on the screen whose position was transformed from the position of the pen. The time from the movement onset and the location of the cursor on the monitor were sampled at 240 Hz. All stimuli were controlled using the Psychophysics Toolbox of MATLAB (MathWorks, Natick, MA, USA).

### Experimental task

The participants performed a go-before-you-know task with two different potential movement distances as the main condition. In the main task, the participants were required to reach and maintain the final cursor on the final target within a time constraint. Participants were randomly divided into two groups based on the time constraints: the group with a time constraint of 500 ms was defined as the fast group (*N* = 11), and the group with a time constraint of 750 ms was defined as the slow group (*N* = 11). Setting a time constraint was important to ensure that the difference in movement distance was reflected in the movement velocity. In this task, the time constraint could be an important variable that defines the possibility of the movement pattern, and the planning policy of the initial movement could be different with changes in the time constraint changes. Thus, we set two conditions of time constraints, and examined whether there would be differences in the strategies for dealing with uncertainty depending on the time constraints.

All potential cursors and targets were circles with a radius of 0.5 cm. There were four possible conditions based on the cursor and target: the *two-target* condition, *two-cursor* condition, *one-target* condition, and *one-cursor* condition. In the *two-target* condition, at the beginning of the task, a cursor (8 cm downward from the center of the screen) and two potential targets (4–12 cm upward from the center of the screen) were presented along a vertical line. In this condition, after the onset of movement, one of the two targets disappeared, and one remained at the initial position. The participants were required to reach and keep the cursor on the remaining target within a time constraint. In the *two-cursor* condition, at the beginning of the task, two potential cursors (4–12 cm downward from the center of the screen) and a target (8 cm downward from the center of the screen) were presented along a vertical line. In this condition, after the onset of movement, one of the two cursors disappeared, and one remained at the initial position. The participants were required to reach and maintain the remaining cursor on the target within a time constraint. The *one-target* and *one-cursor* conditions were the control conditions of the *two-target* and *two-cursor* conditions, respectively. In these conditions, at the beginning of the task, a cursor and a target were presented in a vertical line. However, the cursor and target positions differed between these conditions. In the *one-target* condition, the cursor position remained the same after fixation (8 cm downward from the center of the screen), and the target appeared at a pseudo-random position (4–12 cm upward from the center of the screen) in the vertical direction. In contrast, in the *one-cursor* condition, the target position appeared in the same place after the fixation (8 cm upward from the center of the screen), and the cursor changed to a pseudo-random position (4–12 cm downward from the center of the screen) in the vertical direction. Thus, the *one-target* condition was similar to the *two-target* condition in the process of determining the position of the target, while the *one-cursor* condition was similar to the *two-cursor* condition in the process of determining the position of the cursor. In each condition, to avoid a large difference in the frequency of occurrence for distances, a pseudo-random number was utilized with a uniform distribution restricted with equal probability of occurrence in the three divided ranges were utilized.

In the first task, the participant moved the cursor (white circle, radius 0.5 cm) to the fixation point (white circle, radius 0.5 cm), which was 8 cm below the center of the screen. The actual hand position was almost under the fixation point. After the fixation was complete, potential targets (blue circle, radius 0.5 cm) and cursors (red circle, radius 0.5 cm) were presented on the screen. The cursor for each trial was presented 4–12 cm downward from the center of the screen. The target was presented 4–12 cm from the center of the screen to the top of the screen for each trial (Fig 1C). The participants were required to initiate the movement as soon as the go signal (sound stimulus, 800 Hz) that signaled movement initiation was heard. Movement onset was detected when the hand moved 0.25 cm from the fixation point. After the movement onset, the final cursor and target were determined. If the participant was able to make the cursor reach the target within the time constraint, the trial was considered successful, and after the trial, the word “Hit!” was shown on the screen. If the trial was considered unsuccessful, the word “Miss!’ was shown on the screen. If the movement onset was earlier than the go signal, the words “Too early!’ were shown on the screen. When the movement onset was more than 500 ms later than the go signal, the words “Too late!” were shown on the screen. The participants were instructed to perform a single movement (i.e., the movement velocity goes to zero once after the movement onset in each trial).

The distance between the cursor and the target was classified as *Long (*18–22 cm), *Middle* (14–18 cm), or *Short* (10–14 cm), starting with the farthest one (the positional relationship is shown in Fig 1C). The probability of appearance of these areas was set to be equal. Thus, in the *one-target* and *one-cursor* conditions, 16 trials each were conducted for the three distance conditions, *Long*, *Middle*, and *Short*. In contrast, the *two-target* and *two-cursor* conditions included six distance conditions, *Long-Long*, *Middle-Middle*, *Short-Short*, *Long-Middle*, *Long-Short*, and *Middle-Short*, each of which was also evaluated over 16 trials. Each set included 72 trials, and participants performed four sets (288 trials in total) for an experimental session after a few dozen practice trials to familiarize themselves with the task. Since the purpose of this practice was to check the flow of the task, it was either self-reported by the participants or when the experimenter judged that they understood the task (approximately 10–20 trials were performed). The conditions were randomized equally for each set. The experiments took approximately an hour.

### Data analysis

The observed data were analyzed using programs written in MATLAB software. The cursor positions (horizontal position: *Xc*(*t*), vertical position: *Yc*(*t*)) at each time point (*t*) were smoothed using a second-order, zero-phase-lag, low-pass Butterworth filter with a cutoff frequency of 8 Hz. Movement onset timing was identified at a distance 0.25 cm away from the start position.

The vertical velocity (***V***_*y*_(*t*)) was calculated at each time point (*t*) by differentiating the time series of the vertical position (*Yc*(*t*)). As the index of the initial movement velocity, we calculated the first-peak velocity and first-peak acceleration by using the “*findpeaks*” function in Matlab. Next, in order to eliminate the effect of individual differences in the range of the initial movement velocity and to examine the changes in the initial movement velocity among conditions in terms of the relative amount of change, the first peak velocity was *Z*-valued in each set (including 72 trials) and defined as *Z*_*IMV*_.

To confirm whether the participants’ initial movement parameters were in accordance with the averaging behavior, we calculated the average initial movement velocity using the data in the *one-target* and *one-cursor* conditions and then calculated the difference between the averaging behavior and the observed behaviors (Δ*Z*_*ave*−*obs*_) in the *two-target* and *two-cursor* conditions. For example, Δ*Z*_*ave*−*obs*_ in *the LS* condition (Δ*Z*_*two*−*ave*_[LS]) is defined as ${\Delta Z}_{two-ave}[LS]=Z_{IMV}[LS]-\frac{\left(Z_{IMV}[L]+Z_{IMV}[S]\right)}{2}$, where [] indicates the distance conditions. The closer the Δ*Z*_*two*−*ave*_ is to zero, the closer it is to the averaging behavior. A negative value indicates that the initial velocity is slower than the averaging behavior, while a positive value indicates that the initial velocity is faster than the averaging behavior. To examine the magnitude of the deviation, we also calculated the difference between the average behavior and the actual behaviors in each corresponding *one-cursor/target* condition (for example, ${\Delta Z}_{one-ave}[L]=Z_{IMV}[L]-\frac{\left(Z_{IMV}[L]+Z_{IMV}[S]\right)}{2}$ and ${\Delta Z}_{one-ave}[S]=Z_{IMV}[S]-\frac{\left(Z_{IMV}[L]+Z_{IMV}[S]\right)}{2}$).

To confirm whether the initial movement velocity in the presence of two potential movements was closer to the movement plan for the closer or farther targets, the Δ*Z*_*two*−*one*_ and the overlap of distributions were calculated. First, the mean and variance of the *Z*_*IMV*_ in each condition were calculated. The Δ*Z*_*two*−*one*_ is a measure to evaluate the difference in the mean *Z*_*IMV*_. The Δ*Z*_*two*−*one*_ both between the *two-cursor*/*target* conditions and the nearer *one-cursor/target* conditions and between the *two-cursor/target* condition and the farer *one-cursor/target* condition were calculated. The overlap probability of the two distributions *A*(*p*,*q*) is defined as follows: $A(two,one)=\int_{-\infty }^{\infty }\min\left[N(x|{(\mu }_{two},\sigma_{two}),N(x|{(\mu }_{one},\sigma_{one})\right]dx$, where *N*(*x*|*μ*, *σ*) is a normal distribution with a mean (*μ*) and sigma (*σ*). The larger the overlap of the distributions, the more similar the distributions. As with the other indicators, for each distance condition, the overlap probability with the *one-cursor/target* condition of the closer target and the overlap probability with the *one-cursor/target* condition of the farer target were calculated.

### Data availability

The data supporting the findings of this study are shown in the (S1–S5 Tables).

### Statistical analysis

We conducted three-way repeated-measures ANOVAs (distance condition [3] ×uncertainty condition [2] ×time constraint group [2]) for the Δ*Z*_*two*−*ave*_. We conducted one-sample t-tests with Bonferroni correction on Δ*Z*_*two*−*ave*_ in each condition to determine whether the initial movement velocity in situations with two potential movement distances followed the averaging behavior. We also conducted three-way repeated-measures ANOVAs (uncertainty condition [2] × distance condition [3] ×one target distance condition [2]) for the Δ*Z*_*two*−*one*_ and overlap probability. Partial *η*^2^ for ANOVA [24] and Cohen’s *d* for post-hoc *t*-tests were used to report effect sizes. The partial *η*^2^ = 0.01, = 0.06, and = 0.14 indicates a small, medium, and large effect, respectively. The Cohen’s d = 0.20, = 0.50, and = 0.80 indicates a small, medium, and large effect, respectively. In all statistical tests, the level of significance was set to *p* < .05.

## Results

The current study focused on the initial movement velocity, which is a motor variable that varies with movement distance and examined whether the averaging behavior is confirmed at the initial movement velocity, and if not, whether a patterned difference exists, such as a slower initial movement velocity being observed across the participants by taking movement costs and other factors into consideration. The trial was classified into three distance ranges (Fig 1C), and the change in the initial movement velocity according to the distance was examined.

**Fig 2:**
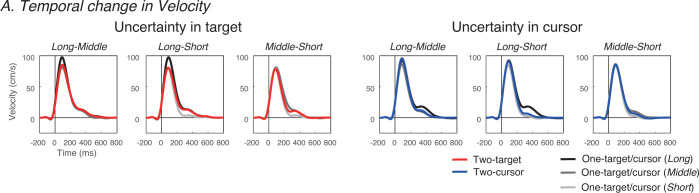
Temporal changes in velocity. Temporal changes in Velocity. These panels show the typical examples of the velocity profile as a function of time in the *Long-Middle*, *Long-Short*, and *Middle-Short* conditions in each *two-potential* condition. Red and blue lines show the *two-potential* conditions. Black, dark-gray, and light-gray lines show the profiles in the *Long*, *Middle*, and *Short* conditions in each *one-potential* condition corresponding to the presented distance conditions of the *two-potential* conditions in each panel. Consistently, as in this participant, velocity profiles with a large first peak were identified.

**Fig 3:**
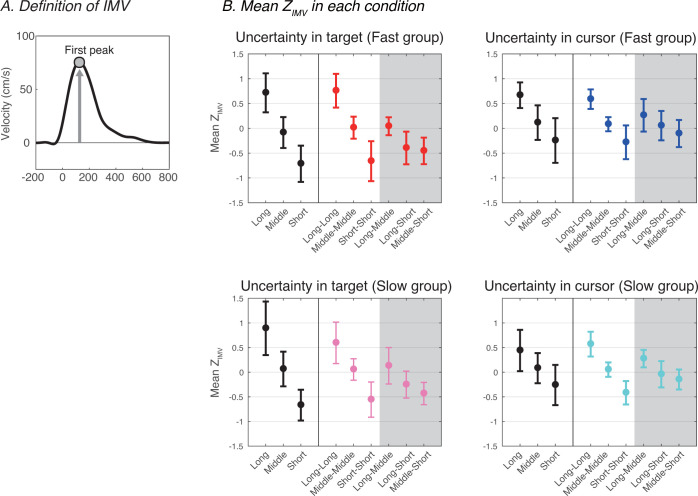
Comparison of mean *Z*_*IMV*_ and mean *Z*_*IMV*_ among conditions. (A) Definition of initial movement velocity (IMV). To offset the differences in velocity ranges between participants, IMV was z-valued within each set (72 trials) for later analysis. (B) Inter-participant means of *Z*_*IMV*_ in each condition. These panels show the inter-participant means and standard deviations of the IMV z-values (*Z*_*IMV*_). Black circles show the data in one-potential conditions (i.e., the *one-cursor* and *one-target* conditions) and other colored circles show the data in two-potential conditions (i.e., the *two-cursor* and *two-target* conditions). The upper row corresponds to the Fast group and the lower row to the Slow group.

Fig 2 shows a typical example of the temporal dynamics of the vertical velocity of the hand in each condition. All participants showed a velocity profile with a large initial peak. To evaluate the change in the initial movement velocity in response to the presented potential difference in movement distance, we detected the first peak of velocity in each condition (Fig 3A) and Z-valued the first peak of speed in each set to offset the variability in the range of velocity changes for each participant. Importantly, paired t-tests revealed that there were no significant differences in *Z*_*IMV*_ between the situation where the near cursor/target was correct and that where the far cursor/target was correct in both two-target and two-cursor conditions (two-target condition: *p* = .657, two-cursor condition: *p* = .739). This suggested that the movements were not corrected according to the correct cursor/target until the timing of the IMV detection.

Fig 3B shows the mean *Z*_*IMV*_ for each group under each condition. These figures suggest that the movement velocity varies depending on the position of the cursor and the target. The data of the *one-cursor/target* conditions suggested that the initial movement velocity changed linearly with the movement distance within the range of the manipulated movement distance. In the *two-cursor/target* conditions, when the two potential targets/cursors were presented at almost the same position (i.e., *Long*-*Long*, *Middle*-*Middle*, and *Short*-*Short*), the initial movement velocity was confirmed to be close to that of the *one-cursor/target* conditions at the corresponding distance. Furthermore, the velocity variation range of the initial phase differed between cases where the cursor had known uncertainty and those where the target had known uncertainty.

**Fig 4:**
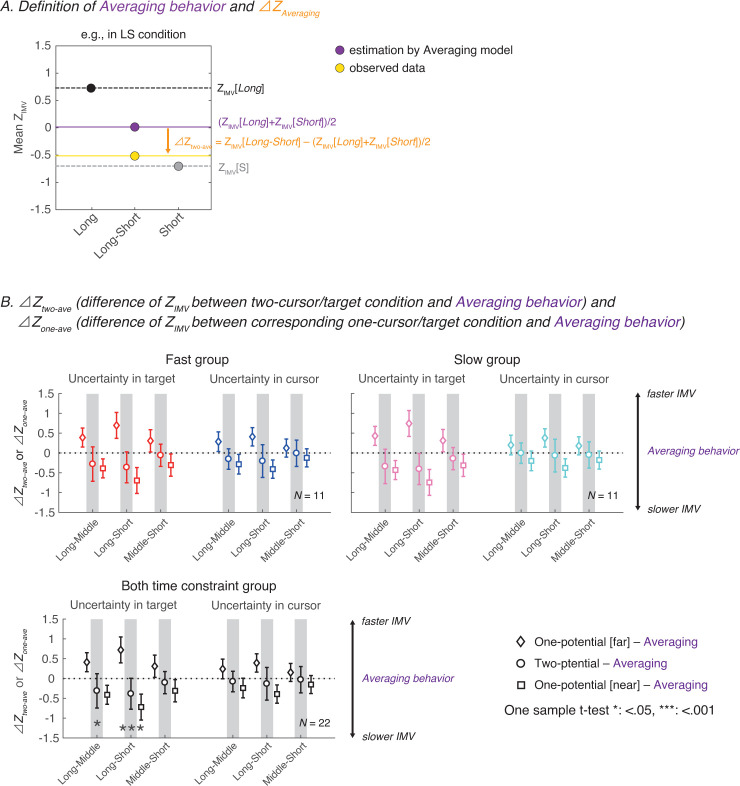
Difference between the estimated averaging behavior and the observed behavior in each condition. (A) Definition of averaging behavior and Δ*Z*_*two*−*ave*_ (difference between the averaging and observed behaviors). The averaging behavior was defined as the average value of *Z*_*IMV*_ in the two corresponding *one-potential* conditions (a purple line and circle). The Δ*Z*_*two*−*ave*_ (an orange arrow) was defined as the difference between the averaging and observed behaviors (a yellow line and circle). (B) Inter-participant means of the Δ*Z*_*two*−*ave*_(circles) and Δ*Z*_*one*−*ave*_(diamonds and squares) in the fast group (upper-left panel), slow group (upper-right panel), and both time constraint group (lower panel) were shown. One-sample *t*-tests with Bonferroni correction on Δ*Z*_*two*−*ave*_ in each condition was conducted using the data pooled from both time constraint group. Besides, one-sample *t*-tests on Δ*Z*_*two*−*ave*_ averaged across distance conditions for each uncertainty condition were conducted to evaluate the overall trend of target uncertainty and cursor uncertainty. The results revealed that the initial movement velocity deviates from the averaging behavior with a patterned bias between participation in *two-target* condition, while the initial movement velocity approached the averaging behavior in the *two-cursor* condition.

In the conditions with a difference in the two potential distances, the findings confirmed that the initial movement velocity was modulated to take into account both movement distances. We tested whether the initial movement velocity at two potential distances followed the averaging behavior. Δ*Z*_*two*−*ave*_ is the difference between the average initial movement velocity of the two discrete actions in the one-distance conditions (i.e., the *one-cursor/target* conditions) and the observed velocity under the *two-cursor/target* conditions (Fig 4A). If the participant performed the average of two discrete actions, then the value of Δ*Z*_*two*−*ave*_ should approach 0. If the motor output is closer to that of a single movement over a short distance, then this value should be negative. Fig 4B shows the inter-participant mean and standard deviation of the Δ*Z*_*two*−*ave*_ and the Δ*Z*_*one*−*ave*_ (circles). To account for the difference in amplitude depending on the movement distance, the difference between the average movement velocity and the movement velocity in the two corresponding *one-cursor/target* conditions (the farer and closer conditions correspond to squares and diamonds, respectively) is also presented for comparison (Fig 4B).

Three-way repeated-measures ANOVA (distance condition [3]×uncertainty condition [2] ×time constraint group [2]) on Δ*Z*_*two*−*ave*_ revealed a significant main effect for distance and uncertainty (distance condition: *F*[2, 40] = 4.374, *η*^2^_*p*_ = 0.179, *p =* .019; uncertainty condition: *F*[1, 20] = 6.812, *η*^2^_*p*_ = 0.254, *p =* .017) and no significant main effect for time constraint group (*F*[1, 20] = 0.021, *η*^2^_*p*_ = 0.001, *p =* .885). There were no significant interactions (distance*uncertainty*time constraint: *F*[2, 40] = 0.149, *η*^2^_*p*_ = 0.001, *p =* .862; distance*uncertainty: *F*[2, 40] = 1.969, *η*^2^_*p*_ = 0.090, *p =* .153; uncertainty*time constraint: *F*[2, 40] = 0.574, *η*^2^_*p*_ = 0.028, *p =* .458; distance*time constraint: *F*[2, 40] = 0.253, *η*^2^_*p*_ = 0.012, *p =* .778). These results indicate the difference in deviations from averaging behavior, depending on the locus of uncertainty and distance of potential actions.

Next, to determine whether the initial movement velocity in situations with two potential movement distances follows averaging behavior, one-sample t-tests compared to 0 (indicating averaging behavior) with Bonferroni corrections for Δ*Z*_*two*−*ave*_ were conducted. Since neither the main effect of time constraint group on Δ*Z*_*two*−*ave*_ no related interaction reached statistical significance, both time constraint groups were pooled as a single condition and one-sample t-tests were conducted for each distance condition (i,e., *Long-Middle*, *Long-Short*, and *Middle-Short*). The findings showed significant differences in *Long-Middle* (*t*[21] = -3.387, *d* = -0.722, *p*_*bonf*_ = .018) and *Long-Short* (*t*[21] = -4.633, *d* = -0.988, *p*_*bonf*_ < .001) in target uncertainty, and no significant differences in *Middle-Short* (*t*[21] = -1.527, *d* = -0.325, *p*_*bonf*_ = .852) in target uncertainty and *Long-Middle* (*t*[21] = -1.228, *d* = -0.262, *p*_*bonf*_ > 1), *Long-Short* (*t*[21] = -1.266, *d* = -0.270, *p*_*bon*_ > 1) and *Middle-Short* (*t*[21] = -0.637, *d* = -0.136, *p*_*bonf*_ > 1) in cursor uncertainty. In addition, to evaluate the overall trend of target uncertainty and cursor uncertainty, one-sample t-tests on Δ*Z*_*two*−*ave*_ averaged across distance conditions for each uncertainty condition were conducted. There was a significant difference in the target uncertainty condition (*t*[21] = − -4.517, *d* = − -0.963, *p*_*bonf*_ < .001), while there was no significant difference in the cursor uncertainty condition (*t*[21] = -1.391, *d* = -0.297, *p*_*bonf*_ = .358). The results show that Δ*Z*_*two*−*ave*_ deviates from the average velocity when two targets are present, with a patterned bias between participation; in contrast, when two cursors were present, the initial movement velocity approached the average value of the single reach movement for each distance. These results suggest that the selection pattern of the initiating action depends on whether uncertainty is present for the target or the cursor.

**Fig 5:**
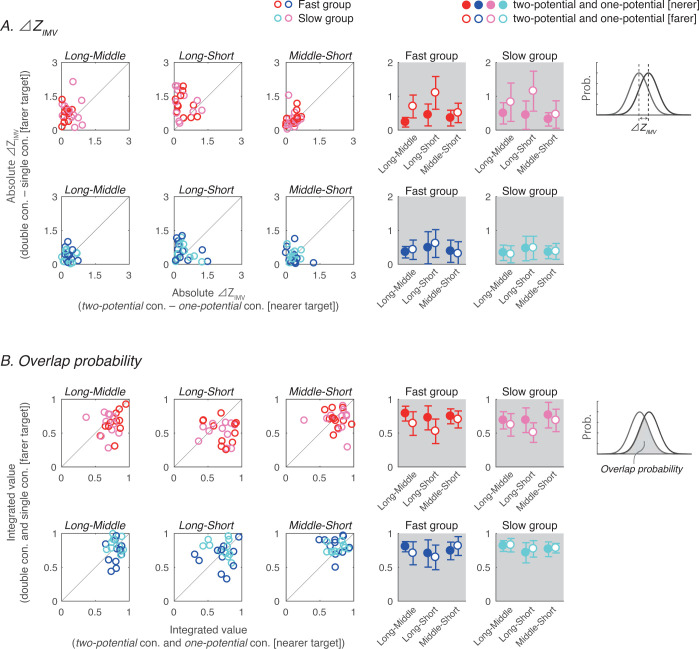
Similarity of the initial movement velocity between the *two-cursor/target* condition and the corresponding *one-cursor/target* condition. (A) Left three panels show the comparisons between Δ*Z*_*two*−*one* [*nearer*]_ and Δ*Z*_*two*−*one* [*farer*]_ in each participant were shown in each distance condition. Right two panels show the comparisons between inter-participant means of Δ*Z*_*two*−*one* [*nearer*]_ and Δ*Z*_*two*−*one* [*farer*]_. (B) Comparison between the overlap probability of distributions of the *two-cursor/target* conditions and the nearer *one-cursor/target* conditions and the overlap probability of distributions of the *two-cursor/target* conditions and the farer *one-cursor/target* conditions. Since the smaller/larger the value of Δ*Z*_*two*−*one*_/overlap probability, the higher the similarity, it was found that the initial movement velocity in the two-target condition was more similar to the one-target condition to the near target. On the other hand, there was no difference in the similarity of the initial movement velocity between the two-cursor and near one-cursor condition, or between the two-cursor condition and far one-cursor conditions.

Next, we present the results for the Δ*Z*_*two*−*one*_ and the probability of overlap of the distributions (Fig 5). Both indices evaluate the similarity between the distributions. These indices can be used to directly evaluate whether the pattern of the initial movement velocity in the condition with two potential distances is similar to either of the movements when each movement is performed in isolation.

Δ*Z*_*two*−*one*_ is an indicator of the difference in the mean *Z*_*IMV*_ between the *two-cursor/target* condition and the corresponding *one-target/cursor* condition. For example, in the *Long-Short* condition, by comparing the difference of *Z*_*IMV*_ between *Long-Short* and *Long* and the difference between *Long-Short* and *Short*, we were able to evaluate which initial movement of the single movement was more similar to that in the *two-cursor/target* condition. A three-way repeated-measures ANOVA (uncertainty condition [2] × distance condition [3] × corresponding one distance condition [2]) on the Δ*Z*_*two*−*one*_ revealed significant main effects of the uncertainty condition (*F*[1, 21] = 14.40, *η*^2^_*p*_ = 0.407, *p* = .001), distance condition (*F*[2, 42] = 23.402, *η*^2^_*p*_ = 0.527, *p* < .001), corresponding one distance condition (*F*[1, 21] = 14.17, *η*^2^_*p*_ = 0.403, *p* = .001), and the interaction effect between the uncertainty and distance conditions (*F*[1, 21] = 3.78, *η*^2^_*p*_ = 0.152, *p* = .031), the interaction effect between the uncertainty and the corresponding one-distance conditions (*F*[1, 21] = 13.40, *η*^2^_*p*_ = 0.389, *p* = .001), and the simple interaction effect between the distance and the corresponding one-distance conditions (*F*[1, 21] = 5.26, *η*^2^_*p*_ = 0.389, *p* = .001). There was no significant two-way interaction effect (*F*[2, 42] = 2.23, *η*^2^_*p*_ = 0.096, *p* = .12). Post-hoc comparisons revealed that the simple main effects of the corresponding one-distance condition for *Long-Middle* (*p* = .002) and *Long-Short* (*p* < .001) in target uncertainty were significant, and the simple main effects of the corresponding one-distance condition for *Middle-Short* (*p* = .072) in target uncertainty and *Long-Middle* (*p* = .839), *Long-Short* (*p* = .609), and *Middle-Short* (*p* = .805) in cursor uncertainty were not significant.

The overlap probability of the distributions takes into account not only the mean but also the variance, and the closer the value is to 1, the higher the similarity between the distributions. A three-way repeated-measures ANOVA (uncertainty condition [2] × distance condition [3] × corresponding one distance condition [2]) of the overlap probability revealed the significant main effects of the uncertainty (*F*[1, 21] = 18.01, *η*^2^_*p*_ = 0.462, *p* < .001), distance (*F*[2, 42] = 12.64, *η*^2^_*p*_ = 0.376, *p* < .001), the corresponding one distance conditions (*F*[1, 21] = 7.37, *η*^2^_*p*_ = 0.26, *p* = .013), and the simple interaction effect between the uncertainty and the corresponding one-distance conditions (*F*[1, 21] = 11.39, *η*^2^_*p*_ = 0.352, *p* = .003). No significant difference was found in the two-way interaction effect (*F*[2, 42] = 2.04, *η*^2^_*p*_ = 0.088, *p* = .143), the simple interaction effect between the distance and the corresponding one-distance conditions (*F*[1, 21] = 2.01, *η*^2^_*p*_ = 0.087, *p* = .147), and the simple interaction effect between the uncertainty and the distance conditions (*F*[1, 21] = 1.35, *η*^2^_*p*_ = 0.06, *p* = .271). Post-hoc tests revealed that the simple main effects of the corresponding one-distance condition were significant in target uncertainty (*p* < .001) and not significant in cursor uncertainty (*p* = .978).

Taken together, the results show that the initial movements in the *two-target* condition are similar to those for a closer target, while the initial movements in the *two-cursor* condition are equally similar to both discrete movements. These results suggest that there is a difference in the strategy for planning the policy of the initial movement depending on whether the known uncertainty is for the target or the cursor.

## Discussion

Previous studies have reported averaging behavior in motor planning in the presence of multiple movement targets [1,2,5,7,8,13–15]. The current study investigated whether the averaging behavior could be confirmed in the planning of the initial movement velocity, taking into account multiple movements with different distances. In addition, we examined whether the planning of the initiation movement velocity would differ depending on whether the uncertainty was in the cursor or in the target. The participants varied the initial movement velocity according to the distance between the cursor and the target. Specifically, in the control conditions (the *one-target* and *one-cursor* conditions), the longer the movement distance, the faster the initial movement velocity. When two potential goal states existed, the initial movement velocity was modulated between the initial movement velocity in the corresponding single movements. However, the behavior differed depending on whether the uncertainty was in the target or in the cursor, such that the initial movement velocity in the *two-target* condition approached that of a single reaching movement toward a nearer target, and the initial movement velocity in the *two-cursor* condition approached the average of the initial movement velocities. These results revealed that in the presence of uncertainty in the cursor, the averaging behavior was confirmed in the planning of the initial movement velocity, while in the presence of uncertainty in the target, a slower movement velocity than the averaging behavior was selected.

### Deviation from the averaging behavior in the *two-target* condition

In the current study, the initial movement velocity in the *two-target* condition was close to the initial movement velocity of a single movement for the closer target. This behavior is consistent with the result that when there are targets with different distances and directions, the action for the target with the closest distance is executed in priority [3,25]. In motor planning where multiple goals exist simultaneously, there is a debate as to whether the weighted average output of discrete motor plans toward each potential target reflects the weighted output or whether it reflects task or cost optimization [16]. The results of the current study do not completely reject either hypothesis, but the important finding obtained herein is that the deviation from the averaging behavior was consistent across participants. In the following paragraphs, we present several possible reasons why a slower movement velocity than the averaging behavior was selected when there were two potential targets.

As mentioned in the Introduction, the first possible explanation is based on the minimization of movement costs. It has been widely confirmed that humans select motor plans that reduce movement costs [26–30], and it is known that humans select a motor target with low costs to reach under multiple targets [25,31]. In addition, previous studies testing motor planning under multiple potential targets suggested that the minimization of movement costs was one of the factors that led to averaging behavior in the initial movement direction [9,32,33]. Moreover, in situations where the averaging behavior does not reflect cost minimization, a deviation to a lower-cost motor plan is observed. Since a sudden deceleration requires a large motor cost, the strategy of planning a movement toward a closer target at the beginning and adding movement distance when necessary seems reasonable.

It is also possible that a smaller initial movement velocity was chosen as a strategy to increase the likelihood of task success. Motor noise is known to increase with movement velocity and distance [34], and a fast movement velocity may increase movement variability [35–37]. Therefore, choosing the minimum movement velocity to reach either target in the beginning may be an effective strategy for increasing the accuracy of reaching the target.

Another possible reason could be the influence of temporal discounting, which prioritizes events that occur earlier in time [38,39]. Humans perceive the value of more immediate rewards to be higher, and temporal discounting has been reported to apply to motor planning [40]. Moreover, in a task with two successive reaching movements, there is a tendency to increase the accuracy of the first reaching movement [41]. Similarly, in the present task, more emphasis may have been placed on reaching the target in the foreground. From these perspectives, it is possible to explain the consequences of selecting a slower initial movement velocity than the averaging behavior.

### Differences in the initial movement planning depending on the characteristics of uncertainty

On the basis of these perspectives, it is possible to explain the consequences of selecting a slower initial movement velocity than the averaging behavior. However, this view is inconsistent with the fact that the average movement velocity of each corresponding single reaching movement was selected under the condition that there were two potential cursors. These results suggest that different control policies are adopted in situations where uncertainty exists in the cursor and those where uncertainty exists in the target.

First, it may be possible to explain the cause of the different control policy wherein movement is planned from the average potential cursor position in the *two-cursor* condition. The gazing point is one possible factor that led to such an integrated process. Since we did not measure eye movements in the present study, we can only speculate, but it is widely confirmed that people generally gaze at the target when performing reaching movements [42,43]. Moreover, visuomotor corrections in response to displacements of the cursor representing the hand position are reported to be fastest and strongest when gaze was directed at the reach target in comparison with those when gaze was directed to a different location in the workspace [44]. For the perception of spatial position, the perception of the cursor position may have been inferior to the perception of the target position because the accuracy of peripheral vision is significantly lower than that of foveal vision [45,46]. Because the spatial uncertainty was higher at the cursor position, the movement may have been planned from an integrated position of the given potential cursors. If so, future research could advance the understanding of motor control mechanisms related to the locus of uncertainty by examining how action selection is modulated by manipulating the gazing point and the time interval from the presentation of a potential cursor or target to the movement onset.

Additionally, the initial movement velocity varied more for changes in the target position than for changes in the cursor position. One of the reasons for this difference may be the effect of the actual hand position. In our experiment, the potential cursor position was presented after the hand was first moved to the fixation point. This procedure may have interfered with motor planning based on the distance from the actual position of the hand to the target, rather than simply motor planning based on the distance between the cursor and the target. In other words, in the conditions where changes occur in the cursor, motor planning based on the coordinate systems of both the center of the potential cursor and the center of the hand may have been represented simultaneously, reflecting the result of their integration. Indeed, the control of movement distance has been reported to be influenced by proprioceptive information as well as the visual location of the effector [23,47–49]. Such a process may also reflect a weighting based on information uncertainty [50,51]. Since the weighting of information varies with the uncertainty of the information, the proprioception weighting could have been higher by performing the movement based on the uncertain visuospatial location for the cursor position, due to gazing at the target in reaching movements in general.

Considering these findings, it is possible that the difference in the control policy of the initial action between conditions does not reflect a difference in the location of uncertainty, but a difference in visual input by gazing points is the determining factor instead. However, in a typical reaching movement, the difference in visual input may be important because the visual input shows obvious disequilibrium between the effector and the target. Future studies will need to examine how somatosensory and visual inputs contribute in an integrated manner to the planning of initial movements by measuring or making explicit the gazing point.

### Effect of time constraints on motor planning of the initial movement velocity

Moreover, although different time constraints were set for the two groups in the current study, the motor planning patterns did not significantly change due to the different time constraints. Since a previous study using the go-before-you-know task reported that when a fast movement velocity is enforced, one target is ignored, it is possible that a more stringent time constraint will cause the target to move toward only one of the targets in the current task setting. In contrast, if a longer time constraint is set, the velocity of movement may be closer to the nearer target when there are two targets at different distances, since there is no advantage in outputting a stronger movement than necessary in advance. Thus, setting a wider range of movement times and considering the modulation of the movement plan according to the movement time may be useful for understanding the sensorimotor control policy. On the other hand, the fact that the characteristics of the motor plans were very similar even when different participants performed the task across groups may indicate that the behavioral patterns in this study were highly consistent across individuals.

### Limitations and scope for future studies

The major limitation of the current study is that it only examined behavioral patterns within a restricted spatial location and time constraint. In future studies, we expect to gain a better understanding of motor planning for multiple potential targets by considering how the initial movements are planned for different distances and different directions along with movement direction and velocity. In addition, it is also important to consider whether the reachability of each target can be accurately determined on the basis of the time constraints and the distance between the target and the cursor and whether this is reflected in the motor plan. These aspects are expected to improve our understanding of higher-order sensorimotor processing of the potential action possibilities. Furthermore, although the motor preparation time was set to be constant in this study, this time may have a significant influence on motor planning and execution. Thus, it is necessary to thoroughly observe how movement patterns change as the spatiotemporal variables of the entire task change, and to construct a theory that can explain this in an integrated manner. Currently, there are a variety of possible explanations for interpreting the observed behavior, and the validity of each hypothesis needs to be examined further.

## Conclusion

The current study investigated (1) whether averaging behavior is confirmed in the initial movement velocity during execution when considering two potential movements with different distances. In addition, the current study examined (2) whether the planning of movement velocity differed depending on the existence of uncertainty in the cursor or in the target. The results revealed that the initial movements in the *two-target* condition were similar to those for a closer target, while the initial movements in the *two-cursor* condition were equally similar to both discrete movements. These results indicate a difference in the strategy for planning the policy of the initial movement depending on whether the known uncertainty is for the movement goal or the effector.

## Supporting information

S1 Table Mean and SD of ZIMV in the two-target and one-target conditions (corresponding to Fig 3B). (PDF)

S2 Table Mean and SD of ZIMV in the two-cursor and one-cursor conditions (corresponding to Fig 3B). (PDF)

S3 Table Mean of ⊿Ztwo-ave (corresponding to Fig 4B). (PDF)

S4 Table Mean of Absolute ⊿Ztwo-one (corresponding to Fig 5A). (PDF)

S5 Table Mean of integrated value (corresponding to Fig 5B). (PDF)
