# Supplementary material for: Different planning policies for the initial movement velocity depending on whether the known uncertainty is in the cursor or in the target: Motor planning in situations where two potential movement distances exist
Source: PLoS One. 2022 Mar 30;17(3):e0265943. doi: 10.1371/journal.pone.0265943 (PMC8967013; doi:10.1371/journal.pone.0265943)
Supplement: S1 Table — (PDF) [file pone.0265943.s001.pdf]

Table 1. Mean and SD of Z_{IMV} in the two-target and one-target conditions (corresponding to Fig 3B).

Two-target and one-target condition																			
		L		M		S		LL		MM		SS		LM		LS		MS	
ID	Group	Mean	SD	Mean	SD	Mean	SD	Mean	SD	Mean	SD	Mean	SD	Mean	SD	Mean	SD	Mean	SD
1	Fast	0.0775	0.7283	-0.0938	1.0371	0.2925	0.8741	0.0356	0.8423	-0.6058	0.9532	-0.1782	1.1587	0.7038	0.9871	0.0929	1.0635	0.1882	1.0064
2	Fast	0.9106	1.0822	-0.4793	0.9678	-0.6657	0.9772	-0.0348	0.8008	-0.0197	0.8400	-0.4907	0.8568	0.3745	0.8495	-0.1694	1.1389	-0.4225	0.8605
3	Fast	0.6641	0.9142	0.1320	0.9964	-0.8216	0.6790	0.3582	0.7223	-0.3603	1.0508	-0.5418	0.8584	0.6454	1.1663	0.3155	0.6141	-0.2705	1.0086
4	Fast	1.2426	0.9568	0.2721	0.7865	-0.8507	0.5139	0.0549	0.9451	-1.0405	0.8580	-0.7709	0.8205	0.5816	0.9863	0.1682	0.9762	0.0717	0.8827
5	Fast	0.7866	0.6821	0.0175	0.9460	-0.9976	0.7545	-0.1420	0.4841	-0.5658	0.6502	-0.6736	0.6212	0.8322	0.9975	-0.0330	1.0779	-0.1140	1.0904
6	Fast	0.4014	0.8325	-0.1860	0.9754	-1.1836	0.8437	-0.2715	0.8212	-0.1794	0.8798	-0.2723	0.9898	0.7434	0.6968	0.1856	0.9621	0.0907	0.9093
7	Fast	0.6581	1.0139	0.0416	0.9107	-0.3179	1.0341	-0.0119	0.9074	-0.2033	0.9333	-0.0028	0.9576	0.3652	1.0750	-0.0252	0.8219	-0.2696	1.1808
8	Fast	0.5247	1.0945	0.2192	0.8341	-0.6497	0.8159	0.0839	0.9058	-0.6319	1.0229	-0.9285	0.8383	0.3075	1.2496	-0.0634	0.5981	-0.4110	0.7329
9	Fast	1.1027	1.0240	-0.1175	0.6223	-0.7417	0.7840	0.2774	0.8221	-0.4900	0.5847	-0.4413	0.7614	0.5532	0.6963	0.0739	0.8788	-0.9545	0.6217
10	Fast	1.0592	1.1600	0.1003	1.1531	-0.9321	0.9228	-0.0615	0.7724	0.1537	0.6665	-0.3707	0.8327	0.8970	0.5940	0.1603	1.0904	-0.3345	0.4960
11	Fast	0.9085	0.8054	0.2365	0.9666	-0.4088	1.2314	0.1704	0.4691	-0.4188	0.9699	-0.3337	0.8608	0.4610	0.7632	0.2071	0.8070	-0.6722	1.1090
12	Slow	0.2404	0.9069	-0.2028	0.8159	-0.4898	0.7053	-0.0791	0.9061	-0.7281	0.9574	-0.6815	0.6243	0.3572	1.0669	0.1675	0.6753	-0.3941	0.9443
13	Slow	0.8232	0.9717	0.1038	0.8174	-1.1075	0.5919	0.2689	0.8962	0.0218	1.2080	-0.6800	0.7109	0.7859	0.8553	-0.1157	0.9969	-0.7495	0.7363
14	Slow	-0.2967	0.9422	-0.2010	0.9451	-0.3445	0.9639	0.3548	1.0103	-0.5323	0.8747	-0.4710	0.7598	0.6621	0.8106	-0.0194	1.3264	0.0433	1.0631
15	Slow	0.8485	0.7136	-0.1213	0.7061	-0.4607	0.9566	0.3135	0.8333	-0.3927	0.8781	-0.1280	1.1695	0.3743	0.8069	0.0800	0.4553	-0.2492	1.0249
16	Slow	0.3099	0.9907	0.0415	0.8392	-0.0502	0.7600	-0.5511	1.0974	-0.3131	0.7528	-0.2905	0.7312	0.6136	0.9098	0.3863	0.8591	-0.4629	0.9815
17	Slow	0.6069	1.1963	0.4979	0.7794	-0.1334	0.9779	0.3363	0.6991	-0.1249	0.9970	-0.5315	0.9276	0.9147	0.6268	-0.0427	0.9616	-0.6053	1.0078
18	Slow	0.8434	0.6607	0.3031	0.6456	-0.4612	1.0785	-0.4193	0.7197	-0.2231	0.9148	-0.0116	1.3098	0.0983	0.8243	0.0395	0.8347	-0.4479	0.7894
19	Slow	0.5287	0.6634	0.0874	0.6830	-0.5897	0.9876	0.5691	0.7916	-0.3601	0.7957	-0.5876	0.9122	0.6721	0.9657	0.1222	0.9016	-0.1744	0.7886
20	Slow	0.8917	0.9632	0.0571	1.0055	-1.0454	0.8126	0.0940	1.0011	-0.0643	1.2497	-0.4309	0.6802	0.4157	0.9457	-0.0564	0.6854	-0.4846	0.6929
21	Slow	1.1633	0.9815	-0.0161	0.7665	-0.8770	0.7007	0.4088	0.7985	0.1995	0.9136	-0.5165	0.5859	0.8033	0.9634	-0.0338	0.7700	-0.6489	0.7152
22	Slow	0.8856	1.1560	0.0954	0.7431	-0.1336	0.7791	0.2400	0.8215	-0.1236	1.1487	-0.0970	0.8706	0.3754	1.0109	-0.0820	0.7734	-1.2096	0.6680
