# Supplementary material for: Different planning policies for the initial movement velocity depending on whether the known uncertainty is in the cursor or in the target: Motor planning in situations where two potential movement distances exist
Source: PLoS One. 2022 Mar 30;17(3):e0265943. doi: 10.1371/journal.pone.0265943 (PMC8967013; doi:10.1371/journal.pone.0265943)
Supplement: S2 Table — (PDF) [file pone.0265943.s002.pdf]

Table 2. Mean and SD of Z_{IMV} in the two-cursor and one-cursor conditions (corresponding to Fig 3B).

Two-cursor and one-cursor condition																			
		L		M		S		LL		MM		SS		LM		LS		MS	
**ID**	**Group**	**Mean**	**SD**	**Mean**	**SD**	**Mean**	**SD**	**Mean**	**SD**	**Mean**	**SD**	**Mean**	**SD**	**Mean**	**SD**	**Mean**	**SD**	**Mean**	**SD**
1	Fast	-0.0410	0.8281	-0.1597	1.2015	-0.3072	1.0028	-0.0933	0.9586	-0.0071	0.9079	-0.1636	0.5962	0.7008	1.2451	-0.2300	0.9634	0.0770	1.0130
2	Fast	0.5333	1.0447	0.5867	0.6675	0.3205	0.7769	0.5104	0.5980	-0.1529	1.0442	-0.7969	0.3654	0.6894	0.2756	0.2804	0.6329	-0.9212	1.1137
3	Fast	0.1902	0.8387	-0.0717	0.9841	-0.2018	0.9683	0.7191	0.9964	0.0282	0.6901	-1.2445	0.7140	0.5680	0.6226	0.5648	0.9328	-0.2579	0.5571
4	Fast	0.3101	0.6765	-0.0552	0.6677	-0.1201	0.7978	0.8550	0.9105	0.3917	0.7607	-1.2908	0.6320	0.9579	0.5202	0.0469	0.5325	-0.2672	0.3762
5	Fast	0.6497	0.9561	0.3574	1.0227	-0.0504	0.7353	0.7794	0.8142	-0.3845	1.1916	-0.6513	0.8400	0.5121	0.6923	-0.1066	0.7996	-0.2791	0.9203
6	Fast	0.1155	0.9670	0.1781	1.0590	0.0804	0.9525	0.6112	0.8391	-0.7651	0.5114	-0.3246	0.9719	0.3313	1.1176	0.5808	0.8827	0.0839	1.0693
7	Fast	-0.0695	0.6672	-0.2340	1.2052	0.0177	1.0857	0.8765	0.6794	-0.2767	0.8419	-0.3809	1.0733	0.9059	0.7021	-0.3744	0.6539	-0.3725	0.9675
8	Fast	0.3467	1.0103	0.2091	0.8418	0.1788	1.0719	0.4048	1.1205	-0.0595	0.7008	-0.4317	0.9162	0.7619	0.8325	0.1639	0.8642	0.6226	0.4671
9	Fast	0.8627	0.7541	0.3130	0.7014	-0.5512	0.6825	0.9877	0.7410	-0.0379	1.5958	-0.8227	0.5331	0.9204	0.8142	0.5544	0.9953	-0.9533	0.5547
10	Fast	0.2320	0.6430	-0.3954	0.7142	-0.5207	0.8788	0.7201	0.6912	0.1067	1.4346	-0.9039	0.6878	0.8466	0.5475	-0.2817	0.7277	-0.0143	0.6893
11	Fast	-0.2411	0.9467	-0.1348	1.0248	-0.0048	0.8352	1.5021	0.8764	0.2187	0.7191	-0.8478	0.6029	0.1435	0.6455	0.0675	1.0291	-0.4220	0.5861
12	Slow	0.5992	0.8986	0.0774	0.9848	-0.2639	0.7994	1.2106	0.7922	0.8400	0.8198	-0.8172	0.6164	1.2363	0.9795	0.3521	0.8789	0.4304	1.1377
13	Slow	0.3885	0.7243	-0.0253	0.5714	0.0138	1.1463	0.8103	0.7367	-0.1979	0.8039	-0.8473	0.6676	0.4003	0.7084	-0.0314	0.6987	0.1893	1.0752
14	Slow	0.1700	0.8724	0.2676	0.9199	-0.2049	1.0893	0.9725	1.3138	0.2914	0.5643	-0.0687	1.0354	0.0596	0.8491	0.2788	0.7227	0.0959	0.6500
15	Slow	0.0571	1.1190	-0.3425	0.9003	0.2379	1.1713	1.3561	0.6975	0.1511	1.0941	-0.5792	1.1527	-0.2427	0.9316	0.0209	0.6594	-0.4347	1.3369
16	Slow	-0.0089	1.0049	0.0453	0.9023	-0.2054	1.2050	-0.0310	1.6143	0.2154	0.9470	-0.5857	0.9011	0.4820	0.8287	0.6776	1.3173	-0.1372	0.6065
17	Slow	0.2068	1.0217	-0.6341	1.2826	0.0180	0.8837	0.2430	0.4715	-0.2792	0.6212	-0.7092	0.6625	0.2135	0.8130	-0.2150	0.8047	-0.3574	0.9059
18	Slow	0.3389	0.7803	-0.0324	1.1882	-0.3573	1.0407	1.6997	1.2785	0.1130	0.6679	-0.4128	0.1986	0.2479	1.3189	0.1979	0.7351	-0.7585	0.4875
19	Slow	0.2842	0.9947	-0.0743	1.0442	-0.2288	1.2360	0.3726	1.3985	-0.3781	0.3724	-0.4882	1.2867	0.7884	1.0770	0.0403	0.8119	-0.5209	1.0342
20	Slow	0.3287	0.9321	0.2078	0.8071	-0.0484	0.7139	1.2191	0.8497	0.0383	0.5948	-1.1165	0.6639	0.7904	0.7100	-0.3800	0.7883	-0.2762	0.8814
21	Slow	0.3887	0.8520	0.0903	0.6832	-0.4426	1.1248	1.0517	0.6950	-0.1498	0.5876	-1.0576	0.7201	0.4291	0.6998	-0.1193	0.9911	-0.8280	0.5155
22	Slow	-0.0199	0.7242	0.5066	1.0552	-0.4449	0.7607	1.1028	0.9793	-0.1205	1.1997	-0.0210	0.6400	0.5888	0.7179	-0.2260	0.8005	-0.7494	0.9641
