# Supplementary material for: Different planning policies for the initial movement velocity depending on whether the known uncertainty is in the cursor or in the target: Motor planning in situations where two potential movement distances exist
Source: PLoS One. 2022 Mar 30;17(3):e0265943. doi: 10.1371/journal.pone.0265943 (PMC8967013; doi:10.1371/journal.pone.0265943)
Supplement: S3 Table — (PDF) [file pone.0265943.s003.pdf]

Table 3. Mean of $\Delta Z_{two\text{-}ave}$ (corresponding to Fig 4B).

$\Delta Z_{two\text{-}ave}$		Uncertainty in target			Uncertainty in cursor		
**ID**	**Group**	**LM**	**LS**	**MS**	**LM**	**LS**	**MS**
1	Fast	0.0858	-0.4774	-0.0928	-0.2764	-0.5486	-0.2307
2	Fast	-0.2135	0.1235	-0.0158	0.0484	0.7026	0.6409
3	Fast	-0.0154	-0.0976	0.0664	-0.3762	-0.2268	-0.3553
4	Fast	-0.5684	-0.8226	-0.3213	-0.1923	-0.4006	-0.0099
5	Fast	-0.3394	-0.6299	-0.1557	0.4470	0.2408	0.1424
6	Fast	-0.1945	-0.3227	0.2726	-0.3405	-0.0295	-0.2519
7	Fast	-0.3117	-0.4511	0.3260	-0.3352	-0.5007	0.3911
8	Fast	-0.0887	-0.6185	-0.6829	-0.1162	-0.4831	-0.2145
9	Fast	-0.1975	-0.5724	-0.0110	0.1252	0.3294	-0.3517
10	Fast	-0.4749	0.2456	0.0279	-0.0505	-0.8116	-0.3727
11	Fast	-0.6900	-0.7460	-0.0192	-0.3466	0.0044	0.1724
12	Slow	-1.1044	-0.9248	-0.6928	-0.1950	-0.7559	-0.6552
13	Slow	-0.0374	0.0403	-0.1574	0.2041	-0.3201	-0.0652
14	Slow	-0.2772	-0.9842	-0.5824	0.0008	0.1899	-0.3922
15	Slow	-0.4401	-0.7811	0.0861	0.1680	-0.0038	0.4448
16	Slow	-0.6433	-0.0047	-0.1053	-0.5887	-0.1272	-0.4756
17	Slow	0.3544	0.1082	-0.0373	0.2076	-0.5621	0.3042
18	Slow	-1.3257	-0.8666	0.1383	0.1160	0.2229	-0.0770
19	Slow	0.5718	-0.3023	-0.1545	-0.1302	-0.2080	0.0115
20	Slow	-0.5347	-0.1156	0.1082	0.1235	-0.0492	0.2797
21	Slow	-0.0421	0.2024	0.0872	0.2338	0.2898	0.0310
22	Slow	-0.2512	-0.6645	-0.0262	-0.2013	0.5868	0.0428
