# Supplementary material for: Different planning policies for the initial movement velocity depending on whether the known uncertainty is in the cursor or in the target: Motor planning in situations where two potential movement distances exist
Source: PLoS One. 2022 Mar 30;17(3):e0265943. doi: 10.1371/journal.pone.0265943 (PMC8967013; doi:10.1371/journal.pone.0265943)
Supplement: S4 Table — (PDF) [file pone.0265943.s004.pdf]

Table 4. Mean of Absolute ⊿$Z_{two\text{-}one}$ (corresponding to Fig 5A).

Absolute ⊿$Z_{two\text{-}or}$		Uncertainty in target						Uncertainty in cursor					
**ID**	**Group**	**LM-M**	**LM-L**	**LS-S**	**LS-L**	**MS-S**	**MS-M**	**LM-M**	**LM-L**	**LS-S**	**LS-L**	**MS-S**	**MS-M**
1	Fast	0.0427	0.1289	0.4422	0.5125	0.0146	0.1711	0.1890	0.7418	0.2367	0.8605	0.3842	0.0772
2	Fast	0.1181	0.5452	0.7771	0.5301	0.3062	0.3378	0.2529	0.1561	1.5079	0.1027	1.2417	0.0402
3	Fast	0.3300	0.3609	0.8842	1.0793	0.7027	0.5699	0.3746	0.3779	0.1862	0.6397	0.0561	0.7666
4	Fast	0.3389	0.7913	0.4044	1.7164	0.5099	1.1586	0.2558	0.6451	0.2082	1.0070	0.1564	0.1579
5	Fast	0.2426	0.9213	0.0855	1.3452	0.0223	0.2891	0.7564	0.1376	0.6364	0.1548	0.2286	0.0562
6	Fast	0.4936	0.8827	0.1452	0.7906	0.0523	0.4929	0.4653	0.2158	0.0942	0.1532	0.0034	0.5004
7	Fast	0.2649	0.8884	0.1776	1.0798	0.3781	0.2740	0.3049	0.9754	0.1385	1.1399	0.3902	0.3921
8	Fast	0.1434	0.3209	0.2002	1.0367	0.4968	0.8690	0.1828	0.4152	0.4134	0.5528	0.4438	0.0149
9	Fast	0.3555	0.7164	0.3145	1.5055	0.3714	0.3766	0.3082	0.0589	1.2748	0.6107	0.4058	1.1126
10	Fast	0.1682	0.7816	1.0576	0.5664	0.5332	0.4773	0.5136	0.6147	0.3811	1.2421	0.5064	0.2391
11	Fast	0.0483	1.3317	0.4290	1.9209	0.5140	0.5524	0.3085	0.3846	0.2872	0.2783	0.4171	0.0723
12	Slow	0.9191	1.2897	0.0891	1.9387	0.1358	1.5214	0.2470	0.6371	0.3530	1.1589	0.6943	0.6161
13	Slow	0.4668	0.5415	0.8691	0.7885	0.1673	0.4821	0.4200	0.0118	0.2146	0.4256	0.1755	0.0452
14	Slow	0.0634	0.6177	0.4636	1.5048	0.4024	0.7625	0.1088	0.1104	0.1718	0.2080	0.3007	0.4837
15	Slow	0.1624	1.0426	0.1866	1.7488	0.4512	0.2791	0.0362	0.2998	0.0921	0.0998	0.6726	0.2170
16	Slow	0.7665	0.5201	0.2726	0.2820	0.2952	0.5058	0.6864	0.4909	0.1825	0.4368	0.0682	0.8830
17	Slow	0.6101	0.0907	0.2424	0.3652	0.2266	0.3147	0.4219	0.0009	0.2786	0.8404	0.3717	0.2328
18	Slow	0.5323	2.1190	0.1897	1.9228	0.4012	0.1246	0.1410	0.0909	0.7261	0.2803	0.4011	0.5552
19	Slow	0.9471	0.1965	0.1281	0.7326	0.0994	0.2095	0.2438	0.5042	0.4466	0.8627	0.2921	0.2691
20	Slow	0.0557	1.1251	1.0522	1.2834	0.6856	0.4692	0.7087	0.4617	0.4841	0.5825	0.2278	0.3315
21	Slow	0.5586	0.6429	1.2571	0.8522	0.5411	0.3667	0.5080	0.0404	0.9183	0.3388	0.3854	0.3233
22	Slow	0.3605	0.8628	0.1026	1.2264	0.0760	0.0236	0.2061	0.6088	1.2559	0.0823	0.3045	0.2189
