# Supplementary material for: Different planning policies for the initial movement velocity depending on whether the known uncertainty is in the cursor or in the target: Motor planning in situations where two potential movement distances exist
Source: PLoS One. 2022 Mar 30;17(3):e0265943. doi: 10.1371/journal.pone.0265943 (PMC8967013; doi:10.1371/journal.pone.0265943)
Supplement: S5 Table — (PDF) [file pone.0265943.s005.pdf]

Table 5. Mean of integrated value (corresponding to Fig 5B).

Integrated-value		Uncertainty in target						Uncertainty in cursor					
**ID**	**Group**	**LM-M**	**LM-L**	**LS-S**	**LS-L**	**MS-S**	**MS-M**	**LM-M**	**LM-L**	**LS-S**	**LS-L**	**MS-S**	**MS-M**
1	Fast	0.9599	0.9194	0.7022	0.7886	0.6897	0.8694	0.8944	0.6791	0.8875	0.7247	0.8488	0.9648
2	Fast	0.8650	0.6781	0.4398	0.6854	0.5799	0.8381	0.7417	0.4314	0.3778	0.5919	0.4966	0.8992
3	Fast	0.8144	0.7856	0.5925	0.5976	0.6483	0.7015	0.8276	0.7641	0.7202	0.6446	0.7379	0.6865
4	Fast	0.8193	0.6694	0.7144	0.3759	0.7076	0.4623	0.8111	0.5774	0.7060	0.3892	0.6591	0.8078
5	Fast	0.5814	0.4516	0.8706	0.3548	0.8547	0.6781	0.6601	0.8349	0.7401	0.8038	0.8538	0.9519
6	Fast	0.6567	0.5949	0.9251	0.6451	0.9776	0.6225	0.7983	0.8975	0.9645	0.9401	0.9441	0.7831
7	Fast	0.8762	0.5636	0.9073	0.4918	0.8459	0.8690	0.8173	0.4760	0.8854	0.5134	0.8429	0.7153
8	Fast	0.8619	0.8462	0.9066	0.6271	0.7744	0.5676	0.8972	0.8067	0.6507	0.7412	0.5778	0.8960
9	Fast	0.7314	0.6458	0.7732	0.2491	0.7355	0.6798	0.8178	0.9566	0.3119	0.6838	0.7344	0.4948
10	Fast	0.7053	0.5913	0.4347	0.6763	0.7166	0.7068	0.7042	0.6014	0.7856	0.3206	0.7308	0.8584
11	Fast	0.7945	0.3010	0.7062	0.2976	0.6937	0.7184	0.8717	0.7505	0.7109	0.7529	0.7297	0.8959
12	Slow	0.5927	0.4458	0.7869	0.2661	0.9127	0.2882	0.8891	0.7324	0.8564	0.5552	0.6897	0.7113
13	Slow	0.7796	0.7299	0.5787	0.6324	0.9004	0.7459	0.7675	0.9878	0.6917	0.7272	0.9322	0.7646
14	Slow	0.7246	0.7669	0.7977	0.4743	0.7815	0.5525	0.8975	0.9478	0.8176	0.9013	0.7294	0.7293
15	Slow	0.8568	0.4922	0.8546	0.2645	0.8458	0.8985	0.7495	0.8614	0.8091	0.9545	0.7826	0.7182
16	Slow	0.7025	0.7727	0.8505	0.6395	0.8335	0.7435	0.7455	0.7765	0.7932	0.7981	0.6792	0.7242
17	Slow	0.6408	0.8004	0.9037	0.6136	0.8996	0.7628	0.7902	0.8990	0.8057	0.6472	0.8329	0.8823
18	Slow	0.6999	0.2730	0.3695	0.3724	0.2730	0.6831	0.9223	0.7500	0.5122	0.9019	0.6109	0.7196
19	Slow	0.3724	0.7253	0.7681	0.6505	0.8324	0.5807	0.8622	0.8052	0.8298	0.6840	0.8732	0.7806
20	Slow	0.7525	0.5397	0.5248	0.5225	0.6099	0.7084	0.6744	0.7549	0.7718	0.6971	0.8556	0.8212
21	Slow	0.6672	0.6629	0.4363	0.5847	0.6697	0.7547	0.7753	0.9034	0.4362	0.8063	0.6101	0.8689
22	Slow	0.7815	0.6270	0.7211	0.5615	0.8531	0.8404	0.8842	0.6734	0.5334	0.8159	0.8294	0.8869
